# Drug Repurposing Applications to Overcome Male Predominance via Targeting G2/M Checkpoint in Human Esophageal Squamous Cell Carcinoma

**DOI:** 10.3390/cancers14235854

**Published:** 2022-11-28

**Authors:** Yin Yin, Xiao Yu, Riyue Feng, Yang Li, Yahui Zhao, Zhihua Liu

**Affiliations:** State Key Laboratory of Molecular Oncology, National Cancer Center, National Clinical Research Center for Cancer, Cancer Hospital, Chinese Academy of Medical Sciences and Peking Union Medical College, Beijing 100021, China

**Keywords:** esophageal squamous cell carcinoma, sex bias, aging, G2/M checkpoint, drug repurposing

## Abstract

**Simple Summary:**

Sex biases in cancer incidence and cancer mortality exist in the majority of cancer types. Esophageal squamous cell carcinoma (ESCC) is a typical malignancy with higher mortality rates and worse responses to treatment in males versus females. To overcome the male predominance in ESCC, more therapeutic targets need to be identified. Meanwhile, age is also an important contributor that should be included when we consider sex bias in cancer. In this study, we used multi-omics data from 663 ESCC patients and found that G2/M checkpoint pathway-related sex bias and age bias were significantly present in genomics, transcriptomics, and epigenomics. Our findings suggest that G2/M targets may be included in combination therapy for male patients to improve the efficacy of ESCC treatment.

**Abstract:**

Esophageal squamous cell carcinoma (ESCC) is strongly characterized by a male predominance with higher mortality rates and worse responses to treatment in males versus females. Despite the role of sex hormones, other causes that may contribute to sex bias in ESCC remain largely unknown, especially as age increases and the hormone difference begins to diminish between sexes. In this study, we analyzed genomics, transcriptomics, and epigenomics from 663 ESCC patients and found that G2/M checkpoint pathway-related sex bias and age bias were significantly present in multi-omics data. In accordance with gene expression patterns across sexes, ten compounds were identified by applying drug repurposing from three drug sensitivity databases: The Connective Map (CMap), Genomics of Drug Sensitivity in Cancer (GDSC), and The Cancer Therapeutic Response Portal (CTRP). MK1775 and decitabine showed better efficacy in two male ESCC cell lines in vitro and in vivo. The drugs’ relevance to the transition between G2 and M was especially evident in male cell lines. In our study, we first validated the sex bias of the G2/M checkpoint pathway in ESCC and then determined that G2/M targets may be included in combination therapy for male patients to improve the efficacy of ESCC treatment.

## 1. Introduction

Sex biases in cancer incidence and cancer mortality exist in the majority of cancer types. Some cancers are typically sex-specific (e.g., ovarian cancer in women and prostate cancer in men). In non-reproductive cancers, men usually showed a higher incidence than women, and nearly twice the mortality rate than women, such as lip cancer, larynx cancer, esophageal cancer, and urinary bladder cancer [1,2]. In addition to differences in cancer incidence and mortality rates, there are also differences in response to treatment between males and females. Females with lung cancer have better survival rates from surgery than males [3]. Moreover, chemotherapy offers a survival advantage to females over males in non-small cell lung cancer (NSCLC), glioblastoma (GBM), and Hodgkin lymphoma (HL) [4,5,6]. At the same time, differences in drug metabolism between sexes frequently result in higher systemic toxicity in female patients [7,8] and influence the efficacy of treatment accordingly. Therefore, understanding the sex bias in responses to cancer treatments is critical for the optimization of treatment approaches to achieve comparable optimal outcomes in both male and female patients. In addition, cancer is a degenerative disease induced by age, whose incidence increases rapidly after approximately the midpoint of life [9]. Sex differences may influence cancer treatment outcomes in different ways, and all effects are modulated by age. For example, adult men have a poorer prognosis and survival than women, which is exacerbated by increasing age, regarding lymphomas [10]. Therefore, when we consider sex bias in cancer, age is a key factor that should be included.

Esophageal cancer is strongly characterized by a male predominance [11]. It ranked as the seventh most common cancer worldwide and the sixth leading cause of cancer death in 2020 [12]. Esophageal cancer can be classified into two subtypes: esophageal adenocarcinoma (EAC) and esophageal squamous cell carcinoma (ESCC). ESCC accounts for 90% of esophageal cancer cases, while approximately 50% of ESCC cases occur in China. The 5-year survival rate of ESCC patients is under 30% [11]. Moreover, one of the major characteristics of ESCC is distinct geographic patterns. In the north-central Taihang Mountain range, which is the most studied region of ESCC in China, the incidence rate of ESCC may exceed 135/100,000 per year [13]. The incidence and mortality rates of ESCC are approximately 2-fold higher in males than in females [12]. Moreover, sex bias in treatment responses also influenced the outcomes of ESCC patients. Radiotherapy treatment of females with ESCC significantly extended their survival compared with males, though at the expense of higher cardiac toxicity [14]. Therefore, to obtain optimal benefit from cancer treatments, the sex of patients should be factored into the development of new strategies for ESCC treatment. Understanding the mechanisms underlying the different cancer vulnerabilities between men and women is fundamental to achieving effective anticancer treatments in ESCC. Previous studies have revealed an association between ESCC and sex hormone receptors such as the androgen receptor (AR) and estrogen receptor β (ERβ) [15,16,17]. However, these studies only partially explained the generally higher incidence of ESCC in men. Studies have not focused on sex bias in the treatment responses of ESCC. Additionally, age was not considered to have a crucial influence on the underlying mechanisms of sex differences in ESCC. Thus, more efforts are required to elucidate the sex bias in ESCC, especially in responses to treatment, with the consideration of age.

One pan-cancer analysis comprehensively characterized the molecular differences in cancer between male and female patients using TCGA datasets [18]. In this study, researchers identified clinically actionable genes as FDA-approved therapeutic targets and corresponding drugs. Moreover, cell line drug screening datasets can be used as another option for more numerous drug discovery applications. The Connective Map (CMap), Genomics of Drug Sensitivity in Cancer (GDSC), and The Cancer Therapeutic Response Portal (CTRP) are the three most widely applied drug sensitivity databases [19,20,21,22,23,24]. These databases link gene expression with drug sensitivity with the goal of accelerating drug repurposing. By building models from drug sensitivity databases and providing the gene expression profiles of patients, clinicians can impute the drug sensitivity score. The application of machine learning models and the development of large data sensitivity databases thus accelerate drug screening. However, no study has applied drug repurposing by bridging the gap between the two groups of patients.

Here, we validated the sex bias in ESCC and examined the biological difference between sexes from the genomics, epigenomics, and transcriptomics of ESCC. In addition, we recognized age-biased genomic instability, and we found that alterations in the G2/M checkpoint caused by aging in females may contribute to the sex bias. Most importantly, by applying machine learning models, we identified two G2/M-related drugs, MK1775 and decitabine, that may be particularly effective in male ESCC patients and may overcome the male predominance in ESCC.

## 2. Materials and Methods

### 2.1. Mutation Signature Analysis

Mutation information in MAF format was read by the Maftools package in R. Pathway diagrams were created by the PathwayMapper [25]. Oncoplots were plotted by choosing genes for which the mutation rate of samples mutated over 0.05. Genes that mutated with sex bias were selected by the Maftools package, and the minimum number of samples mutated in at least one of the cohorts was four. The somatic interaction plots were also generated by the Maftools package, and pairwise Fisher’s exact test was performed to detect mutually exclusive or co-occurring events [26]. All plots were calculated by the Maftools package and were generated by the ggplot2 package [27].

### 2.2. CNV

The distribution of cytobands and the number of genes they included and the percentage of samples included were calculated in R, and the plot was generated by ggplot2 [27]. The segments and recurrent copy number gains or losses across samples were generated and plotted using the GISTIC 2.0 algorithm [28]. The circular overview of amplification and deletion in both sexes was generated by the circlize package [27].

### 2.3. Differentially Methylated Region (DMR)

DMRs were calculated by the methodyKit package in R with a 5000 bp tilling window size and 5000 bp step size. The minimum number of bases to be covered in a window was set as 10. The significant DMRs were identified by the MethodyKit package with a *p*-value < 0.05 [29]. All DMR CpGs and gene regions were annotated by the annotatr package [30]. The basis for CpG-related annotations is the CpG island tracks from the AnnotationHub package, and the basis for genic annotations is from the TxDb.Hsapiens.UCSC.hg19.knownGene packages [31]. Hg19 was used as the genome assembly. The gene region annotation included the following regions: 1–5 kb upstream of TSSs, promoters, 5UTRs, exons, introns, and 3UTRs. The CpG annotation included CpG islands, shores, shelves, and interCGI regions. The density plot, gene parts percentage plot, and distribution plot of significant gene methylation levels between tumor and normal tissues and between sexes were generated by ggplot2 [27].

### 2.4. Drug Sensitivity Database

GDSC2 (Genomics of Drug Sensitivity in Cancer), CTRP (The Cancer Therapeutics Response Portal), and CMap (Connectivity Map) were used in drug prediction.

#### 2.4.1. Genomics of Drug Sensitivity in Cancer (GDSC)

GDSC is part of a Wellcome-funded collaboration between The Cancer Genome Project at the Wellcome Sanger Institute (UK) and the Center for Molecular Therapeutics, Massachusetts General Hospital Cancer Center (USA). GDSC can be used to inform the optimal clinical application of cancer drugs and has significant effects on the design, cost, and ultimate success of new cancer drug development [19]. Here, our analysis included the drug sensitivity data of 805 cell lines and screened them with 198 compounds from GDC2.

#### 2.4.2. The Cancer Therapeutics Response Portal (CTRP)

The Cancer Therapeutics Response Portal (CTRP) was developed by researchers at the Center for the Science of Therapeutics at the Broad Institute and is sponsored in part by the NCI’s Cancer Target Discovery and Development Network. CTRP connects the genetic features of cancer cell lines to small-molecule sensitivity to accelerate the discovery of patient-matched cancer therapeutics [20,21,22]. Here, our analysis included the drug sensitivity data of 829 cancer cell lines to 545 compounds from CTRP2.

#### 2.4.3. Connectivity Map (CMap)

CMap is funded by the NIH LINCs (Library of Integrated Cellular Signatures) projects. The library of CMap contained over 1.5 M mRNA expression profiles from ~5000 small-molecule compounds and ~3000 genetic reagents screened in multiple cell types. A series of web applications are available for researchers to access and manipulate the data [23,24].

### 2.5. Drug Repurposing Analysis

OncoPredict is an R package for predicting in vivo or cancer patient drug response and biomarkers from cell line screening data. Two models were built by using GDSC2 and CTRP2 separately as training data [32]. The drug sensitivities were predicted by those two models, and our expression data of the top 3000 were differentially expressed in the two sexes as input data. T-tests were performed between the two sexes. CMap’s Query web application was used for finding the perturbagens that give rise to similar or opposing expression signatures. Unfortunately, the sex-biased drugs predicted by the three databases do not overlap because the drug sensitivity data covers different areas and the number of included drugs is different. Therefore, we filtered the drugs based on the publications, *p*-values in the t-test, and drug scores from CMap. Based on the selection results of the three databases, ten drugs were selected for the following validation.

### 2.6. Drug-Target Network

STRING is a database of known and predicted protein–protein interactions. The interactions in STRING are derived from five main sources: genomic context predictions, high-throughput lab experiments, (conserved) coexpression, automated text mining, and previous knowledge in databases. The STRING database currently covers 24,584,628 proteins from 5090 organisms. The drug-target network was constructed by the STRING database [33].

### 2.7. Gene Set Variation Analysis

Gene set variation analysis (GSVA) was implemented by the GSVA package in R. GSVA enrichment scores were estimated by setting the minimum and maximum sizes of the resulting gene sets to 3 and 100,000 [34].

### 2.8. Correlation Expression and Protein Level

The correlation between the gene expression level and protein level was performed in R and plotted by ggplot2.21.

### 2.9. Reagents

Decitabine (HY-A0004), MK1775 (HY-10993), Lapatinib (HY-50898), Brefeldin. A (HY-16592), OSI.027 (HY-10423), SB-216763 (HY-12012), IB-MECA (HY-13591), Tandutinib (HY-10202), Parbendazole (HY-115364), and 5. Fluorouracil (HY-90006) were purchased from MedChemExpress (MCE, NJ, USA).

### 2.10. Cell Culture

The human ESCC cell lines KYSE30, KYSE150, KYSE450, and KYSE510 were generously provided by Dr. Yutaka Shimada. Cells were cultured in RMPI 1640 medium supplemented with 10% FBS. KYSE30 and KYSE450 cells were derived from male patients, while KYSE150 and KYSE510 cells were derived from female patients.

### 2.11. IncuCyte Cell Proliferation Assay

KYSE30 cells were seeded into 96-well plates at a density of 2500 cells per well. KYSE150, KYSE450, or KYSE510 cells were seeded into 96-well plates at a density of 3000 cells per well. After adherence, the cells were treated with drugs at the indicated concentrations and photographed every 3 h with IncuCyte S3 (SINSITECH, Beijing, China). Cell proliferation was measured using the metric of phase object confluence (%).

### 2.12. Xenograft Transplantation Experiments

A total of 1 × 10^6^ KYSE30 cells or 3 × 10^6^ KYSE450 cells were subcutaneously injected into 6-week-old male Balb/c nude mice (GemPharmatech Co., Ltd., Nanjing, China). A total of 1 × 10^6^ KYSE150 cells were subcutaneously injected into 6-week-old female Balb/c nude mice (GemPharmatech Co., Ltd., Nanjing, China). The tumor size was measured every other day once the tumor reached 25 mm^3^. The tumor volume was calculated as 0.5 × length × width^2^. After the tumors reached 25 mm^3^, the mice were randomly divided into vehicle or drug groups. MK1775 (60 mg/kg, p.o.) or decitabine (1.0 mg/kg, i.p.) was administered daily or once every two days until sacrifice. Mice were sacrificed 2 weeks after drug administration, and tumors were dissected and weighed. The animal experimental procedures were approved by the Animal Care and Use Committee of the Chinese Academy of Medical Sciences Cancer Hospital.

### 2.13. RNA-Sequencing Assay

RNA-sequencing analysis was performed by Novogene (Beijing, China). Briefly, KYSE30 and KYSE150 cells were treated with MK1775 (200 nM) for 36 h or decitabine (10 μM) for 48 h. Three pairs of samples were generated from three biologically independent experiments. mRNA was purified from total RNA using magnetic beads with oligo-dT and fragmented randomly. The first strand and the second strand of cDNA were then synthesized using reverse transcriptase and DNA Polymerase I. The reverse transcription product was converted into blunt ends, followed by adenylation of the 3′ ends. Subsequently, the DNA fragments were ligated with an Illumina universal adapter. After PCR amplification, the PCR product was purified by AMPure XP beads (Beckman Coulter, Beverly, CA, USA) and quantified by an Agilent 2100 Bioanalyzer and Qubit 2.0 Fluorometer. The prepared libraries were then sequenced by the Illumina NovaSeq 6000.

### 2.14. Differential Expression Analysis

Differentially expressed genes between tumor and normal samples and between sexes were identified using the DESeq2 package [35]. Only genes with a *p*-value below 0.05 and log2 Fold above 1 were included. In validation RNA-seq, differentially expressed genes between normal and after treatment were identified using the DESeq2 package as well. The heatmap plot was generated by the ComplexHeatmap package [36].

### 2.15. Reactome Pathway Analysis

Reactome pathway analysis was performed by the ReactomePA package [37]. All Reactome plots were generated by the GOplot and enrichplot packages [38,39]. All enrichments were identified with an adjusted *p*-value cutoff below 0.05 and a q-value above 1.

## 3. Results

### 3.1. The Cell Cycle, WNT, and RTK/RAS/PI3K Pathways Contribute to Sex-Biased and Age-Biased Mutation Rates

Our data included mutation information in tumors and paired normal tissues from 663 ESCC patients (Table 1). There were 388 patients whose age was over or equal to 60 years old. Among them, 256 patients were male, while 132 patients were female. In addition, there were 275 patients whose age was below 60 years old. Among them, 182 patients were male, while 93 patients were female.

**Figure 1:**
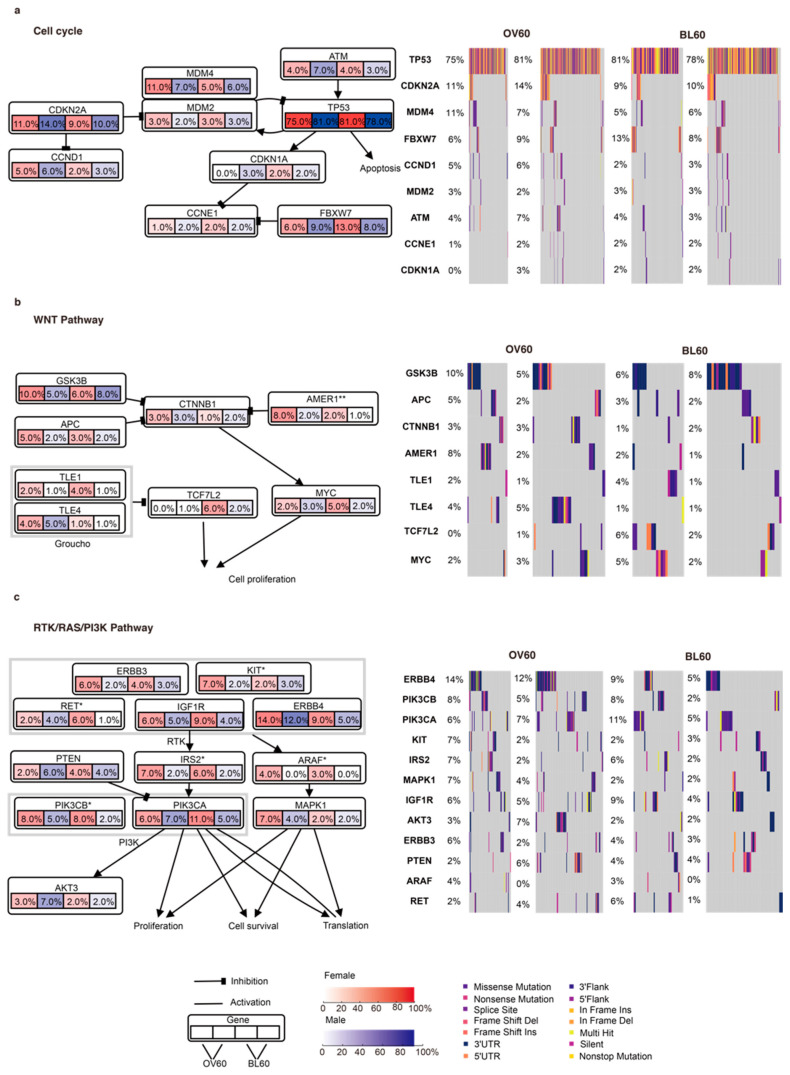
Integrated comparison of somatic mutations between sexes and between age groups. Genes are grouped by three different pathways: (**a**) cell cycle, (**b**) WNT, and (**c**) RTK/RAS/PI3K. Genes that showed significant differences between sexes are labeled. Data were analyzed using the fisher test on a 2 × 2 contingency. (* *p* < 0.05 and ** *p* < 0.01). Red shading denotes female and blue shading denotes male. The color level indicates the mutation rate level, while arrows and lines show the gene interactions in the pathway. In each gene box, the left two numbers indicate the mutation rates of the two sexes in the over 60 age group, while the right two numbers indicate the mutation rates of the two sexes in the below 60 age group.

The cell cycle, WNT, and RTK/RAS/PI3K pathways showed a sex-biased mutation rate (Figure 1). In the cell cycle pathway, the difference in mutation rate between sexes in *TP53*, *CDKN2A*, *ATM*, and *MDM4* was larger in elderly individuals (Figure 1a). At the same time, the mutation rates of *CDKN2A*, *MDM4*, and *CCND1* in both sexes in the over 60 age group were higher than those in the below 60 age group. Moreover, the difference in the mutation rate of *FBXW7* was larger in the group younger than 60 years. In the WNT pathway, *AMER1* (*p* = 0.006) showed a great mutation rate difference between sexes in the over 60 age group (Figure 1b). The *GSK3B* and *APC* genes tended to have larger mutation rate differences between sexes in the over 60 age group, while the *TCF7L2* and *MYC* genes tended to have larger mutation rates between sexes in the below 60 age group. Furthermore, *TCF7L2* showed a higher mutation rate in younger people, while *TLE4* showed a higher mutation rate in elderly people.

In the RTK/RAS/PI3K pathway, the *KIT* (*p* = 0.048), *ARAF* (*p* = 0.004), and *IRS2* (*p* = 0.013) mutation rates between sexes were significantly different in the over 60 age group but not in the below 60 age group (Figure 1c). Moreover, *ERBB3*, *PTEN*, *MAPK1*, and *AKT3* presented a larger sex bias in the mutation rate in the over 60 age group. Additionally, *PIK3CB* (*p* = 0.034) and *RET* (*p* = 0.02) presented significant opposite trends, which were significantly different mutation rates between sexes in the younger than 60 years age group. At the same time, although not significant, *PIK3CA* and *IGF1R* showed similar results. Importantly, regardless of sex, *ERBB4* and *MAPK1* had a higher mutation rate in the over 60 age group.

The top 50 mutated genes in the four subgroups (OV60: Female, OV60: male, BL60: Female, and BL60: Male) showed different rankings (Figure S1a–d). The mutation rate in all top 50 genes was beyond 5%. Among the four subgroups, the OV60 male group had the highest mutation rate, 80%. *TP53* and *TTN* had the two highest mutation rates in tumors from all genes in all four subgroups. In the OV60 age group, males showed a generally higher mutation rate in tumors than females (Figure S1a). In the BL60 age group, the gene compositions of the top 50 mutated genes were similar. In addition, in the OV60 age group, *EPPK1* (*p* = 0.044), *KMT2C* (*p* = 0.044), *DNAH8* (*p* = 0.048), *TENM1* (*p* = 0.013), and *KALRN* (*p* = 0.041) had significantly higher mutation rates in females, while *APOB* (*p* = 0.039), *CACNA1E* (*p* = 0.026), *COL3A1* (*p* = 0.041), *ASPM* (*p* = 0.024), and *OTOGL* (*p* = 0.041) had significantly higher mutation rates in males (Figure S2a). The missense mutation was the most common variant classification in all nine genes in the OV60 age group (Figure S2b,g). Among the ten genes, *COL3A1* and *APOB* presented high co-occurrence (*p* = 0.017). In addition, *DNAH8* and *KMT2C* showed similar patterns (*p* = 0.031) (Figure S2c). In the BL60 age group, *UNC13C* (*p* = 0.034), *BRCA2* (*p* = 0.008), *HDAC6* (*p* = 0.008), *F8* (*p* = 0.019), and *GPR112* (*p* = 0.020) had significantly higher mutation rates in females, while *AJUBA* (*p* = 0.031), *RIF1* (*p* = 0.031), *TRPM6* (*p* = 0.031), *VWA3B* (*p* = 0.031), and *ZNF536* (*p* = 0.031) had significantly higher mutation rates in males (Figure S2d). Similar to the OV60 age group, missense mutation was the most common variant classification in all ten genes in the BL60 age group (Figure S2e,h). Furthermore, *GPR112* and *UNC13C* relationships co-occurred (*p* = 0.002) (Figure S2f). Mutated genes with sex bias in the OV60 age group had a higher mutation rate than those in the BL60 age group.

### 3.2. Male Patients over 60 Years Old Have More CNV Events

**Figure 2:**
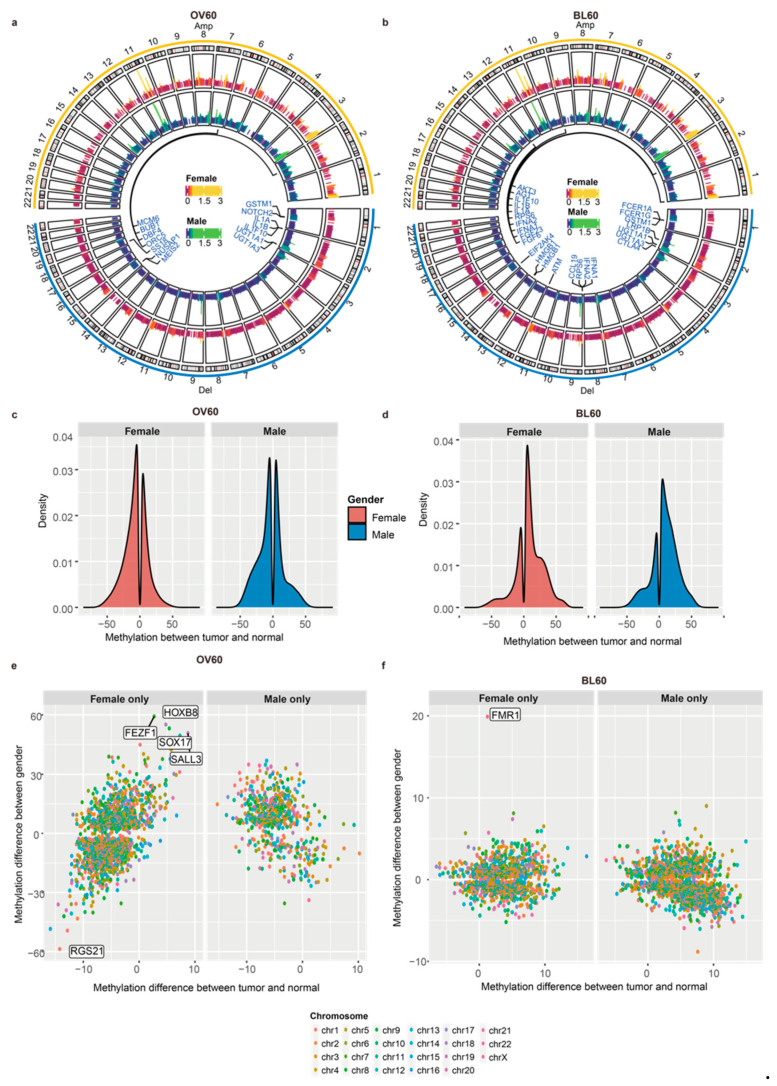
Male patients over 60 years old had more CNV regions and more sex-biased methylation in promoter regions. (**a**) CNV circular genome visualization of patients over 60 years old and (**b**) patients below 60 years old. Genes related to the G2/M phase transition were annotated. (**c**) Patients over 60 years old and (**d**) patients below 60 years old. A density plot of methylation difference levels between tumors and normal tissues. (**e**) The methylation level distribution of patients over 60 years old and (**f**) patients below 60 years old. The y-axis shows the methylation difference level between sexes, while the x-axis shows the methylation difference level between tumors and normal tissues.

In the OV60 age group, the top three cytobands with the highest number of genes in each sex were the same: 11q13.3, 2q22.1, and 9p21.3 (Figure S3a). In the BL60 age group, the top three cytobands in males were 11q13.3, 11q13.4, and 9p21.3, while the top three cytobands in females were 11q22.1, 11q13.3, and 9p21.3 (Figure S3b). Although the top three cytobands were not the same top three cytobands, most were still in the same chromosome. The OV60 age group and BL60 age group shared two cytobands, 9p21.3 and 11q13.3, in the top three cytobands. We also found amplification and deletion cytobands in each sex. In both age groups, most amplifications occurred on chromosome 11 and chromosome 3, while most deletions occurred on chromosome 9 (Figure 2a,b). Among all genes in significant copy number variation (CNV) regions, genes related to the G2/M phase were annotated. From the OV60 age group, both amplification and deletion in males had more locations than those in females. Six amplifications shared the same cytoband between sexes: 3q28, 8p11.21, 8p24.21, 11q13.3, 12q15, and 14q13.3, and three deletion locations shared the same cytoband between sexes: 1p13.2, 2q22.2, 4q35.2, and 9p21.3 (Figure S3c). From the BL60 age group, both genders had a similar number of cytobands in amplification and deletion. Additionally, most cytobands were shared by the two sexes. Regarding the amplified cytobands, 11q13.3, 11q22.1, 3q28, 7p11.2, and 7q21.2 overlapped between the sexes. Regarding the deleted cytobands, 9p21.3, 4q35.2, 2q22.1, and 8p23.2 overlapped between the sexes (Figure S3d). By comparing the OV60 and BL60 age groups, we showed that the OV60 age group had a greater number of CNV events and more sex differences.

### 3.3. Elderly Individuals Have More Sex-Biased Methylation Levels in Promoter Regions

We compared the overall differentially methylated region (DMR) density between the two age groups (Figure 2c,d). For each age group, the methylation pattern between the sexes was similar, while the methylation pattern between age groups was different. Regarding the difference between age groups, in the OV60 age group, the number of DMRs in the tumor was similar to that in the normal group, while more DMRs occurred in tumors in the BL60 age group.

The distribution of gene parts and CpG locations under hypomethylation or hypermethylation is presented (Figure S3e,f). Methylation locations were separated into two categories: 1. Only occurred in females and 2. Only occurred in males. In the OV60 age group, the intergenic region had the highest percentage among all gene parts, while the inter-region between CpG islands had the highest percentage among all CpG regions. Both hypomethylation and hypermethylation shared a similar percentage in all regions. There was no significant sex difference between females and males (Figure S3e). In the BL60 age group, the intergenic region had the highest percentage among all gene parts, while the inter-region between CpG islands had the highest percentage among all CpG regions. The percentages of intergenic regions, introns, and intro-exon boundaries differed between promoter regions that only occurred in females and promoter regions that only occurred in males.

The distribution of promoter regions with methylation levels between tumors and normal tissues and methylation levels between sexes in the two age groups is also presented (Figure 2e,f). We split DMRs that have different methylation levels between normal tissues and tumors into two groups: female only and male only. In the female only group, DMRs only occurred in female patients and did not occur in male patients. In the male only group, DMRs only occurred in male patients and did not occur in female patients. Regarding age, the methylation level in the normal samples was higher in older people, while the methylation level in the tumor samples was higher in younger people. In addition, compared with younger people, older people had more genes with sex-biased methylation levels.

In the OV60 group, DMRs in the female only group were more numerous than DMRs in the male only group. DMRs that only occurred in female patients had more sex-biased methylation levels. Among DMRs that only occurred in female patients, *RGS21* was more easily methylated in males while *FEZF1*, *HOXB8*, *SOX17*, and *SALL3* were more easily methylated in females than other promoter regions (Figure 2e). In addition, in the female only group, there was a trend that DMRs methylated in the tumor were more easily methylated in females. Among these genes, SRY (sex determining region Y)-box 17 (*SOX17*) is a transcription factor important for esophagus tissue development and is hypermethylated in human cancers [40]. Moreover, the promoter hypermethylation of *SOX17* correlates with poor chemoradiation therapy response in ESCC and *SOX17* overexpression sensitizes the response through the downregulation of DNA repair genes or damage response genes [41]. In the male only group, DMRs that easily methylated in females were more numerous than DMRs that easily methylated in males.

In the BL60 age group, the distribution of most promoters shared similar patterns between sexes. *FMR1* was the only gene with a methylation level between sexes above 10, which suggests that *FMR1* is more easily methylated in females (Figure 2f).

### 3.4. G2/M Checkpoint Phase Contributes to Sex Bias and Age Bias at the Same Time

**Figure 3:**
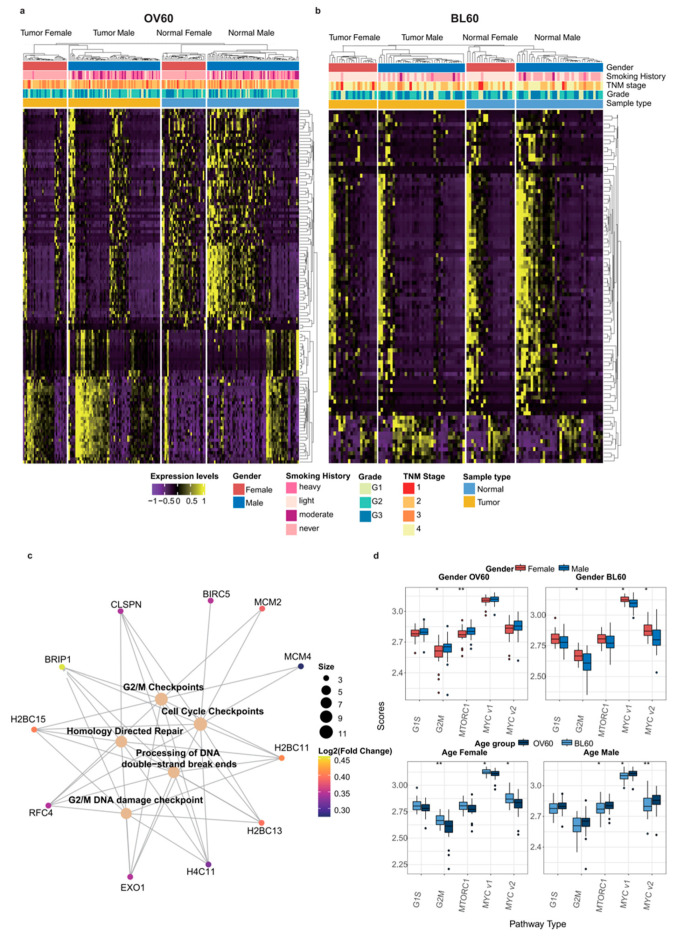
The G2 M phase contributes to sex disparity and age disparity at the same time. (**a**) DEGs in patients over 60 years old and (**b**) patients below 60 years old. (**c**) The Gene-Concept network in the OV60 age group between Reactome pathways achieved by pathway enrichment analysis and their related genes in DEGs. The log2FC of each gene is color scaled, and the size of the gene sets of each pathway is also labeled by the size of the circle. (**d**) GSVA scores calculated comparison in the four different groups: (1) Gender OV60: compares the scores between genders only in samples over 60 years old; (2) Gender BL60: compares the scores between genders only in samples below 60 years old; (3) Age Female: compares the scores between age groups only in female samples; (4) Age Male: compares the scores between age groups only in male samples. Gender and age groups are labeled with colors. Data were analyzed using the Wilcoxon test. (* *p* < 0.05 and ** *p* < 0.01).

Differential gene expression analysis between the sexes is shown by age group (Figure 3a,b). Compared with the BL60 age group, gene expression in the OV60 age group was more dissimilar between the sexes and between the tumors and normal tissues. In the OV60 age group, the separation between genes and the separation between normal tissues and tumors were clear. Regardless of normal samples or tumor samples, the expression patterns of the two genders were different.

In the OV60 group, pathway enrichment analysis results revealed that genes with significant sex differences were mainly related to the G2/M DNA damage checkpoint and homology-directed repair. Among all genes that contributed to those pathways, *BRIP1*, *H2BC11*, *H2BC13*, *H2BC15*, *H4C11*, and *RFC4* were shared among most pathways (Figure 3c). In both age groups, the G2/M pathway showed sex differences. In addition, *MTORC1* is another sex-biased pathway in patients over 60 years old, while *MYC1* and the *MYC2* pathways had sex-biased activity between the sexes in patients below 60 years old. Moreover, *MYC1* and *MYC2* were also age-dependent pathways in both female and male patients. Interestingly, the G2/M pathway was only age-dependent in female patients (Figure 3d).

### 3.5. Decitabine and MK1775 Show Sex-Biased Drug Sensitivity

**Figure 4:**
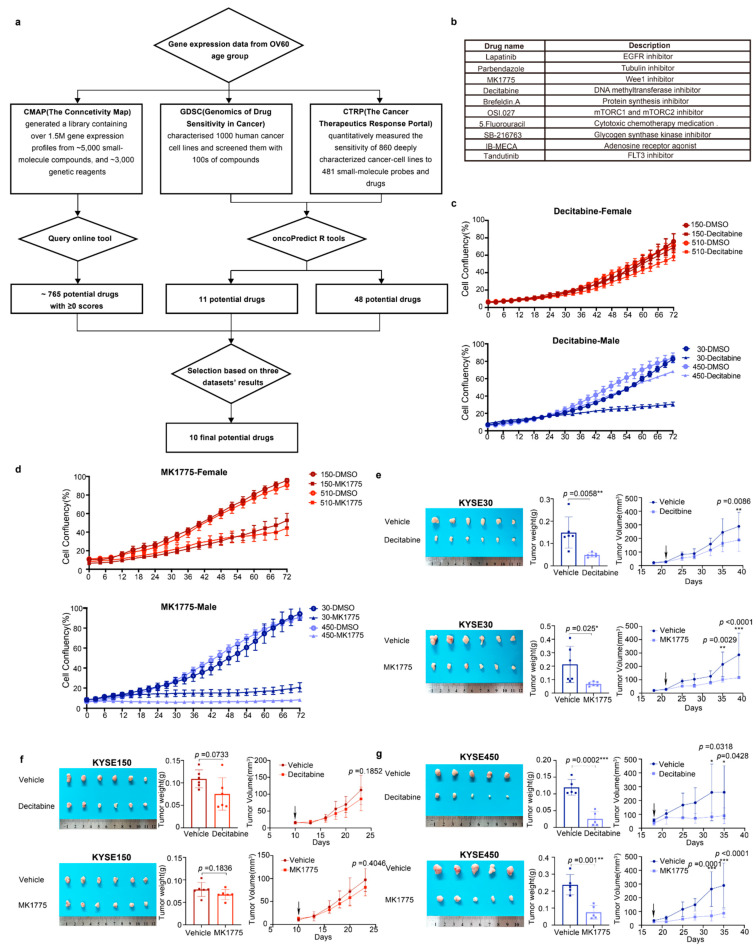
Male ESCC cell lines were more sensitive to decitabine and MK1775 than females. (**a**) Workflow of drug repurposing by using the gene expression data of patients over 60 years old. (**b**) Ten drugs were selected from the drug repurposing results. (**c**,**d**), Growth curves of KYSE150, KYSE510, KYSE30, and KYSE450 cells were measured by IncuCyte S3 for 72 h. KYSE30 and KYSE450 cells were derived from male patients, while KYSE150 and KYSE510 cells were derived from female patients. Cells were treated with decitabine (10 μM) or MK1775 (200 nM). (**e**–**g**), Representative image, tumor weights, and tumor volumes of xenografts derived from KYSE30 cells (male), KYSE150 cells (female), and KYSE450 (male) that were treated with decitabine (1.0 mg/kg, i.p.) or MK1775 (60 mg/kg, p.o.). The data shown are the mean ± SD; *n* = 6 mice per group in KYSE30 and KYSE150; *n* = 5 mice per group in 450. For tumor weights, data were analyzed using two-tailed t-tests; for tumor volumes, data were analyzed using two-way ANOVA with Bonferroni correction. (* *p* < 0.05, ** *p* < 0.01, and *** *p* < 0.001; *ns* = not significant).

The workflow of drug prediction is shown in Figure 4a. We first chose three drug sensitivity databases: CMap (The Connective Map), GDSC (genomics of Drug Sensitivity in Cancer), and CTRP (The Cancer Therapeutic Response Portal). By using the Query in CMap, we selected 765 potential drugs with scores greater than 0 (Table S1). Since GDSC and CTRP do not have such an online tool, we applied the machine learning process only to GDSC and CTRP datasets. The machine learning process was performed using the oncoPredict R package. We used the GDSC2 and CTRP2 datasets as training data and used the expression data of the top 3000 differently expressed drug sensitivities in the two sexes as input data. Then, we built a model using ridge regression. The oncoPredict will predict the IC50 for each drug and each patient. In order to identify the sex-biased drugs, we performed a t-test between the two sexes (Tables S2 and S3). Unfortunately, the sex-biased drugs predicted by the three databases do not overlap because the drug sensitivity data covers different areas and the number of included drugs is different. Therefore, we filtered the drugs based on the publications, *p*-values in the *t*-test, and drug scores from CMap. Based on that, we finally selected ten drugs that might potentially have significant sex-biased sensitivity to ESCC patients (Figure 4b).

We then screened the ten candidate drugs with cell proliferation assays using the male-derived ESCC cell lines KYSE30 and KYSE450 and the female-derived ESCC cell lines KYSE150 and KYSE510. Among the ten drugs, decitabine and MK1775 were identified to have a stronger inhibitory effect on male-derived ESCC cell lines (Figure 4c,d). The other eight drugs exhibited similar inhibitory efficiency in male and female ESCC cell lines (Figure S4).

To further validate the antitumor effect of decitabine and MK1775 on male and female ESCC in vivo, we xenografted male and female ESCC cell lines subcutaneously into male and female BALB/c nude mice. When the tumor volume of xenografts reached ~25 mm^3^, the mice were randomly separated into the drug or vehicle group. MK1775 (60 mg/kg, p.o.) or decitabine (1 mg/kg, i.p.) was administered until the mice were sacrificed. Consistent with the in vitro experimental results, the male-derived ESCC cell lines KYSE30 and KYSE450 showed lower growth rates in vivo and smaller tumor volumes of xenografts than the female-derived ESCC cell line KYSE150 (Figure 4e–g). Collectively, MK1775 and decitabine showed sex-biased drug sensitivity in ESCC cell lines with greater sensitivity in males.

### 3.6. The G2/M Pathway Contributes to Sex-Biased Drug Sensitivity

**Figure 5:**
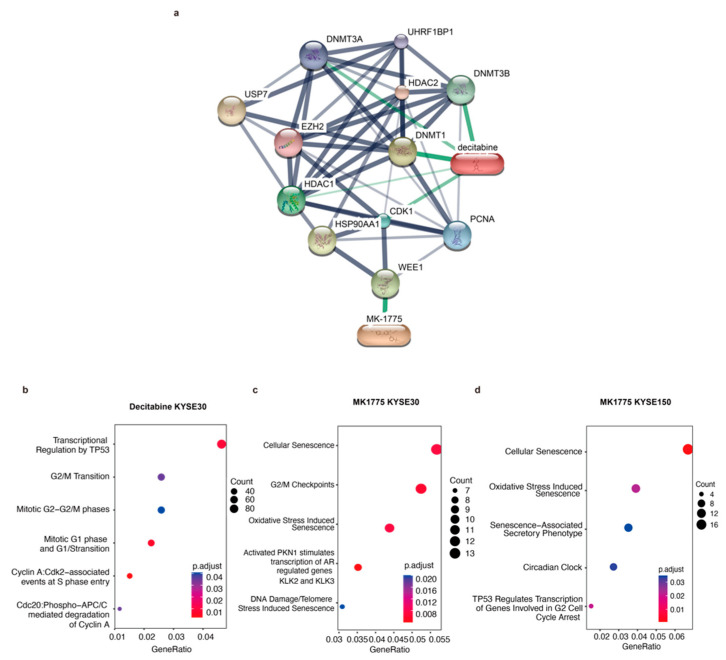
Validation RNA-seq proves that MK1775 and decitabine showed a sex-biased treatment response by targeting the G2/M checkpoint. (**a**) Drug-gene network between decitabine and MK1775 and their target genes was assessed in STICH. Gene functional ontology analysis in the (**b**) decitabine KYSE30 cell line, (**c**) MK1775 KYSE30 cell line, and (**d**) MK1775 KYSE150 cell line.

In the drug-gene network between decitabine and MK1775 and their target genes, both drugs were tightly related to CDK1, which plays a key role in the control of the eukaryotic cell cycle by modulating the centrosome cycle as well as the mitotic onset and promoting the G2-M transition (Figure 5a). As mentioned above, the G2/M pathway showed a difference in males and females. To validate our hypothesis, validation RNA-seq experiments were performed (Figure 5b,d). However, there were not many differentially expressed genes between decitabine treated and untreated KYSE150 cells. Therefore, there was no ontology annotation, which can also reflect the low sensitivity of the KYSE150 cell line to decitabine. The pathways that decitabine simultaneously influences include the G1/S transition and G2/M transition (Figure 5b). Moreover, after MK1775 treatment, cellular senescence and G2/M checkpoints were influenced. Most importantly, an androgen-dependent pathway was significantly enriched in the male KYSE30 cell line but not the female KYSE150 cell line after MK1775 treatment (Figure 5c,d). Thus, we demonstrated that the different effects of decitabine and MK1775 on male and female ESCC cell lines were associated with the G2/M pathway.

## 4. Discussion

Although significant sex bias occurs in cancer incidence as well as mortality and treatment responses, aside from the role of sex hormones, other causes that may contribute to sex bias in ESCC remain largely unknown, especially because the hormone difference between the sexes is lost with age. Our study demonstrated the contribution of the G2/M checkpoint pathway to sex bias from the transcriptomics, genomics, and epigenomics level. Moreover, by evaluating a total of ~3500 drugs from in vitro drug screening data in three databases, The Connective Map (CMap), Genomics of Drug Sensitivity in Cancer (GDSC), and The Cancer Therapeutic Response Portal (CTRP) [19,20,21,22,23,24], and utilizing machine learning models, we predicted the drug response of older females and males. By comparing the predicted drug responses between the sexes, we selected 10 drugs that may have sex bias in elderly individuals. After validation in vitro and in vivo, MK1775 and decitabine showed their potential to overcome sex bias in elderly individuals.

Genome instability is a critical sign of aging and is a hallmark of cancer [42]. Genome stability is supported by a complex system of DNA repair, damage tolerance, and checkpoint pathways. With aging, a series of accumulated damages, such as UV radiation, X-rays, and chemicals, will be brought into DNA. Therefore, DNA stability is a major target of aging [43]. Genomic instability can be shown in mutation, CNV, and epigenetics. In our work, age bias was presented in mutation, CNV, methylation, and gene expression data. Patients over 60 years old had a higher mutation rate and had more CNV regions at the same time, which indicated DNA instability in older patients due to aging (Figure S1 and Figure 2). In addition, aging is more commonly associated with CpG hypomethylation. Loss of CpG methylation also causes genomic instability and increases the risk of cancer [44]. In this study, when patients were older, the methylation level started to diminish, which may lead to tumor development (Figure 2c).

Additionally, sex bias was more evident in older ESCC patients. Here, we investigated sex biases in two age groups of ESCC multi-omics data. Including mutation, CNV, and gene expression, the differences between the sexes were more significant in elderly individuals. Changes in hormonal secretory patterns occur during aging, including decreased estrogen production in older women and decreased testosterone levels in older men [45]. With these changes, the G2/M checkpoint pathway starts to contribute to the sex difference in patients over 60 years old. More importantly, the G2/M checkpoint pathway also contributed to the difference between age groups. Cell cycle arrest is a critical characteristic for the identification of all types of senescence. This phenomenon is caused by a continuing shortening of telomeres upon each cell division and represents a physiological response to prevent genomic instability and therefore accumulation of DNA damage [46]. Furthermore, the impact of the G2/M checkpoint on cancer induction has been reviewed previously [47]. Therefore, cell cycle arrest is closely related to cancer and aging through cellular senescence. In our study, females had a significant difference in the G2/M checkpoint pathway between the over-60-year-old age group and the below-60-year-old age group, while males did not (Figure 3d). Hence, compared with males, females tend to have more genetic instability brought on by aging, which leads to a larger sex bias in elderly individuals.

Moreover, our findings highlight that decitabine and MK1775 may overcome sex bias in drug sensitivity through the G2/M checkpoint phase. Decitabine is an inhibitor of DNA methyltransferases, and it induces cell cycle arrest at the G2/M phase and apoptosis in human cancer cells [48]. Decitabine was thought to be incorporated into DNA, replacing cytosine in the DNA, and covalently trapping DNA methyltransferase into DNA, leading to irreversible inhibition of DNA methyltransferase [49]. A previous study showed a sex difference in CDA expression and its impact on decitabine treatment outcomes, which also demonstrated that sex is an influential factor in the response to decitabine [50]. Furthermore, as older people have more genes with sex differences in methylation levels (Figure 2e), decitabine may be more effective in elderly male patients. MK1775 is a WEE1 inhibitor and has been reported to be included in clinical trials on DNA-damaging therapies in various cancer types [51]. WEE1 is a kinase involved in cell cycle regulation and the DNA damage response and was also identified as a synthetic lethal partner specific to *ATRX* deficiency in cancer [52]. The role of the G2/M checkpoint is to prevent cells from initiating mitosis when DNA damage occurs [53]. The G2/M transition is mainly regulated by the phosphorylation status of cyclin-dependent kinases (CDKs). Before mitosis, CDK1 is maintained in an inactivated state by WEE1 through phosphorylation of CDK1 at tyrosine 15, and CDK1 is then phosphorylated at threonine 14 by myelin transcription factor (MYT1). Hence, WEE1 acts as a negative regulator of entry into mitosis at the G2/M transition. Enrichment analysis results of our RNA-sequencing data also revealed that differentially expressed genes in male ESCC cell lines treated with MK1775 were more associated with G2/M checkpoints. At the same time, decitabine treatment induced variations in both the G2/M and G1/S phase transitions. This finding can be explained by the targeting of MK1775 to the G2/M checkpoint, while decitabine is a relatively broad-spectrum inhibitor of methyltransferases. Collectively, our findings highlighted the sex bias in the regulation of G2/M checkpoint-related genes in ESCC. In addition to MK1775 as a WEE1 inhibitor, other candidate G2 checkpoint abrogators were investigated as a means of enhancing the therapeutic index of cytotoxic agents [54]. Therefore, G2/M targeted therapy may overcome sex bias and optimize treatment approaches. Additionally, the study emphasizes the need to consider sex as a variable in both basic science and clinical research.

Chemotherapy, including cisplatin, fluorouracil, and paclitaxel, remains one of the standard treatments for ESCC patients. At the same time, immunotherapy, such as anti-PD-L1 therapy, has been reported to have promising efficacy in ESCC [55,56]. Programmed cell death ligand 1 (PD-L1) is a protein that helps cancer cells escape death by T cells. PD-L1 inhibitors are considered to be targeted drugs to expose cancer cells to the immune system [57]. Common PD-L1 inhibitors used in ESCC are pembrolizumab and camrelizumab. Previous clinical studies have shown immunotherapy efficacy in ESCC [58,59]. Meanwhile, it has been reported that there is a different immunotherapy response between sexes in ESCC and other types of cancers [60,61,62]. However, the mechanism behind the sex bias in the immunotherapy response remains unclear.

Decitabine and MK1775 have sex-biased efficacy in ESCC cell lines and may be considered potential drugs for combination therapy in the future to overcome the sex bias in immunotherapy response. Additionally, the changes in the immune tumor environment after applying these two drugs need to be further investigated. Since CD8^+^ T-dependent antitumor immunity for mediating sex differences in tumors has been mentioned in one previous study [63], more research needs to be conducted on humoral immunity and the influences of utilizing these two drugs.

## 5. Conclusions

Except for the role of sex hormones, other causes that may contribute to sex bias in ESCC remain largely unknown, especially as the hormone difference between the sexes is lost with age. Our study demonstrated the contribution of the G2/M checkpoint pathway to sex bias in elderly patients from the transcriptomics, genomics, and epigenomics level. Decitabine and MK1775 have sex-biased efficacy in ESCC cell lines and may be considered potential drugs for combination therapy in the future to overcome the sex bias in immunotherapy response. Additionally, the changes in the immune tumor environment after applying these two drugs need to be further investigated. More research needs to be conducted on humoral immunity and the influences of utilizing these two drugs.

## Figures and Tables

**Table 1:** Clinical information of ESCC patients.

Characteristic	Female (*n* = 225)	Male (*n* = 438)
Age		
≥60 years	132 (58.7%)	256 (58.4%)
<60 years	93 (41.3%)	182 (41.6%)
Smoking History		
heavy	2 (0.9%)	104 (23.7%)
light	1 (0.4%)	26 (5.9%)
moderate	7 (3.1%)	182 (41.6%)
never	215 (95.6%)	126 (28.8%)
Tumor Grade		
G1	21 (9.3%)	46 (10.5%)
G2	146 (64.9%)	287 (65.5%)
G3	58 (25.8%)	105 (24.0%)
TNM stage		
Stage I	22 (9.8%)	31 (7.1%)
Stage II	121 (53.8%)	230 (52.5)
Stage III	78 (34.6%)	163 (37.2%)
Stage IV	4 (1.8%)	14 (3.2%)

Data are presented as number (%).

## Data Availability

Data can be accessed in the Genome Sequence Archive (GSA) in the BIG Data Center (http://bigd.big.ac.cn/gsa, accessed on 1 October 2021), Beijing Institute of Genomics (BIG), Chinese Academy of Sciences, with the BioProject number PRJCA004501 and accession number HRA000021. The code can be accessed at https://github.com/LexiYin11/ESCC_sex_bias (accessed on 1 September 2022).

## Supplementary Materials

The following supporting information can be downloaded at: https://www.mdpi.com/article/10.3390/cancers14235854/s1, Figure S1: Mutation landscape in different sex groups and age groups; Figure S2: Sex-biased mutated genes in the over 60 age group and below 60 age group; Figure S3: CNV and methylation in different sex groups and age groups; Figure S4: Proliferation rate of eight other candidate drugs that potentially have sex-biased sensitivity to ESCC patients; Table S1: CMap drug scores; Table S2: T test results between two sexes in drug scores calculated by using CTRP2 as train data; Table S3: T test results between two sexes in drug scores calculated by using GDSC2 as train data.
