# Supplementary material for: Drug Repurposing Applications to Overcome Male Predominance via Targeting G2/M Checkpoint in Human Esophageal Squamous Cell Carcinoma
Source: Cancers (Basel). 2022 Nov 28;14(23):5854. doi: 10.3390/cancers14235854 (PMC9741366; doi:10.3390/cancers14235854)
Supplement: Supplementary file 1 [file cancers-14-05854-s001.zip › Supplementary Information.pdf]

Drug repurposing applications to overcome male predominance via targeting G2/M checkpoint in human esophageal squamous

Yin Yin[#], Xiao Yu[#], Riyue Feng, Yang Li, Yahui Zhao[*], Zhihua Liu[*]

State Key Laboratory of Molecular Oncology, National Cancer Center, National Clinical Research Center for Cancer, Cancer Hospital, Chinese Academy of Medical Sciences and Peking Union Medical College, Beijing 100021, China.

[#]Y.Y. and X.Y. contributed equally to this work.

[*]Correspondence: liuzh@cicams.ac.cn (Z.L.); zhaoyh@cicams.ac.cn (Y.Z.)

Supplementary Information

Supplementary Figures

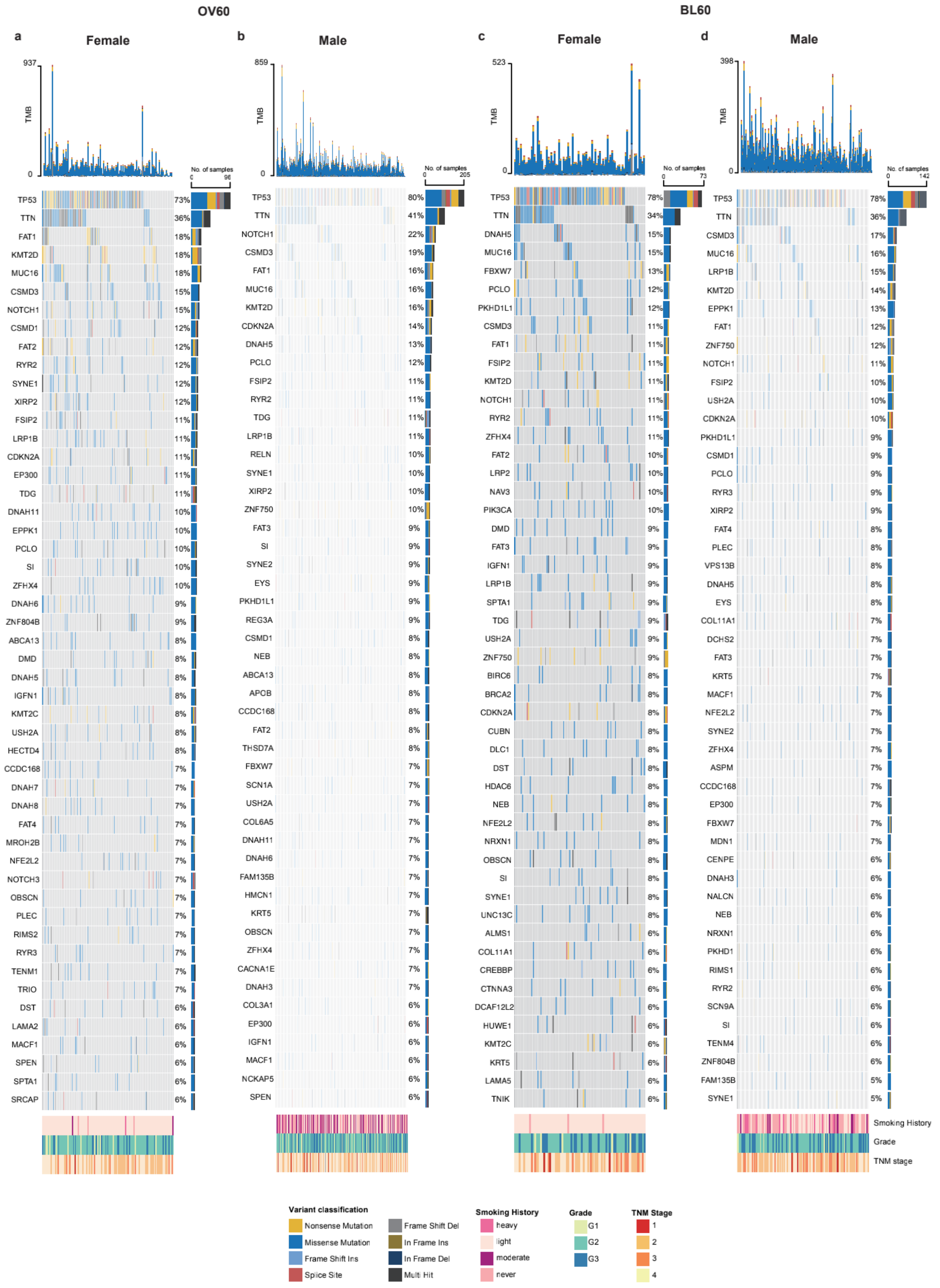

Figure S1 Mutation landscape in different sex groups and age groups. (a) and (b) Female and male top 50 mutated genes in patients over 60 years old. (c) and (d) Female and male's top 50 mutated genes in patients below 60 years old. Patients shared a

similar gene composition in the top 50 mutated genes. The mutation ratios in all top 50 genes were greater than 5%.

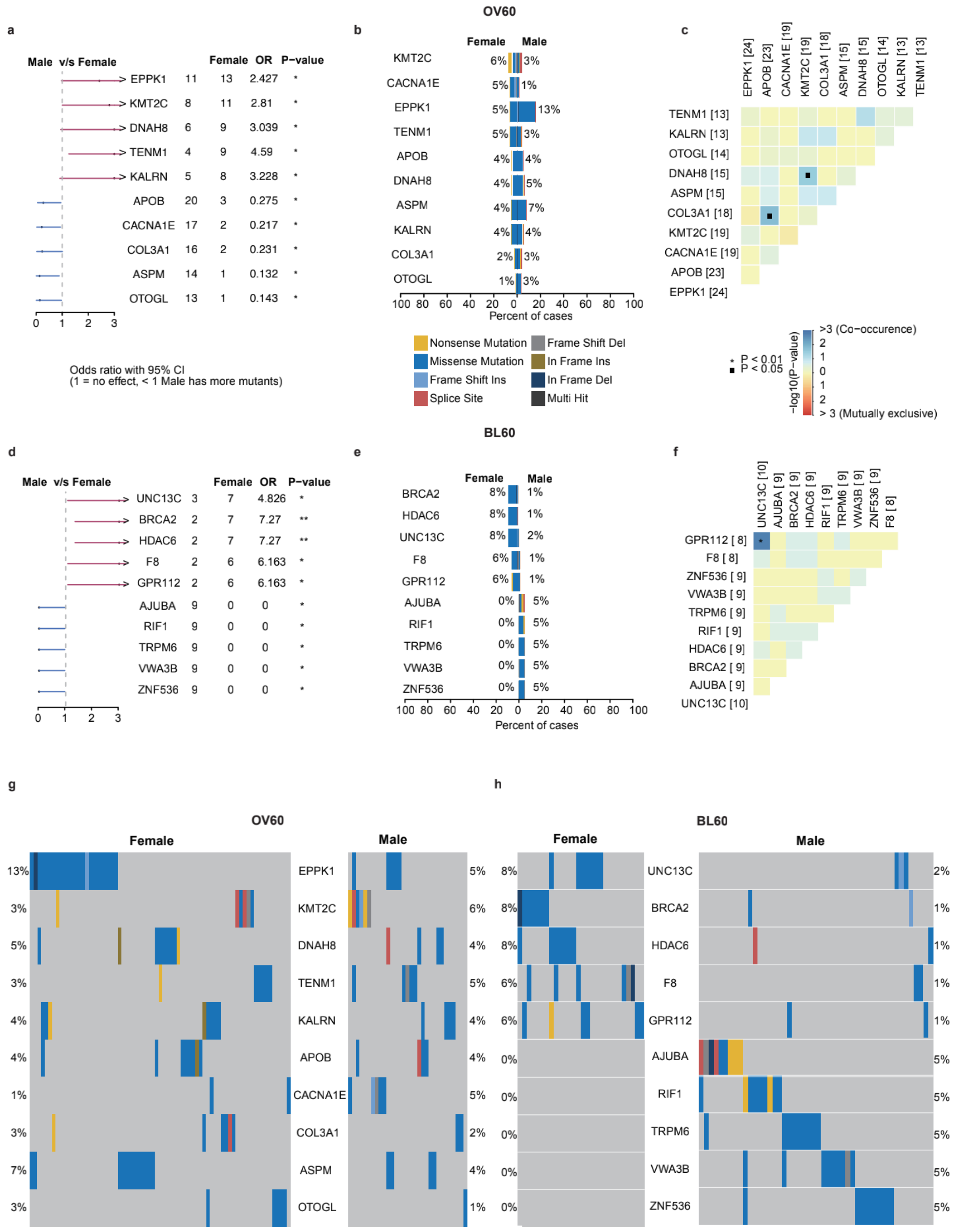

Figure S2 Sex-biased mutated genes in the over 60 age group and below 60 age group. (a) Patients over 60 years old and (d) patients below 60 years old's forest plot of patients over 60 years old between genders. (b) Patients over 60 years old and (e)

patients below 60 years old's variant classification co-bar plot between sexes. (c) Patients over 60 years old and (f) patients below 60 years old's somatic mutation interaction plot between nine genes shown in (a) and (d). (g) Patients over 60 years old and (h) patients below 60 years old's onco-plot between genders. Data were analyzed using fisher test on 2x2 contingency table. (*$p < 0.05$, **$p < 0.01$, and ***$p < 0.001$)

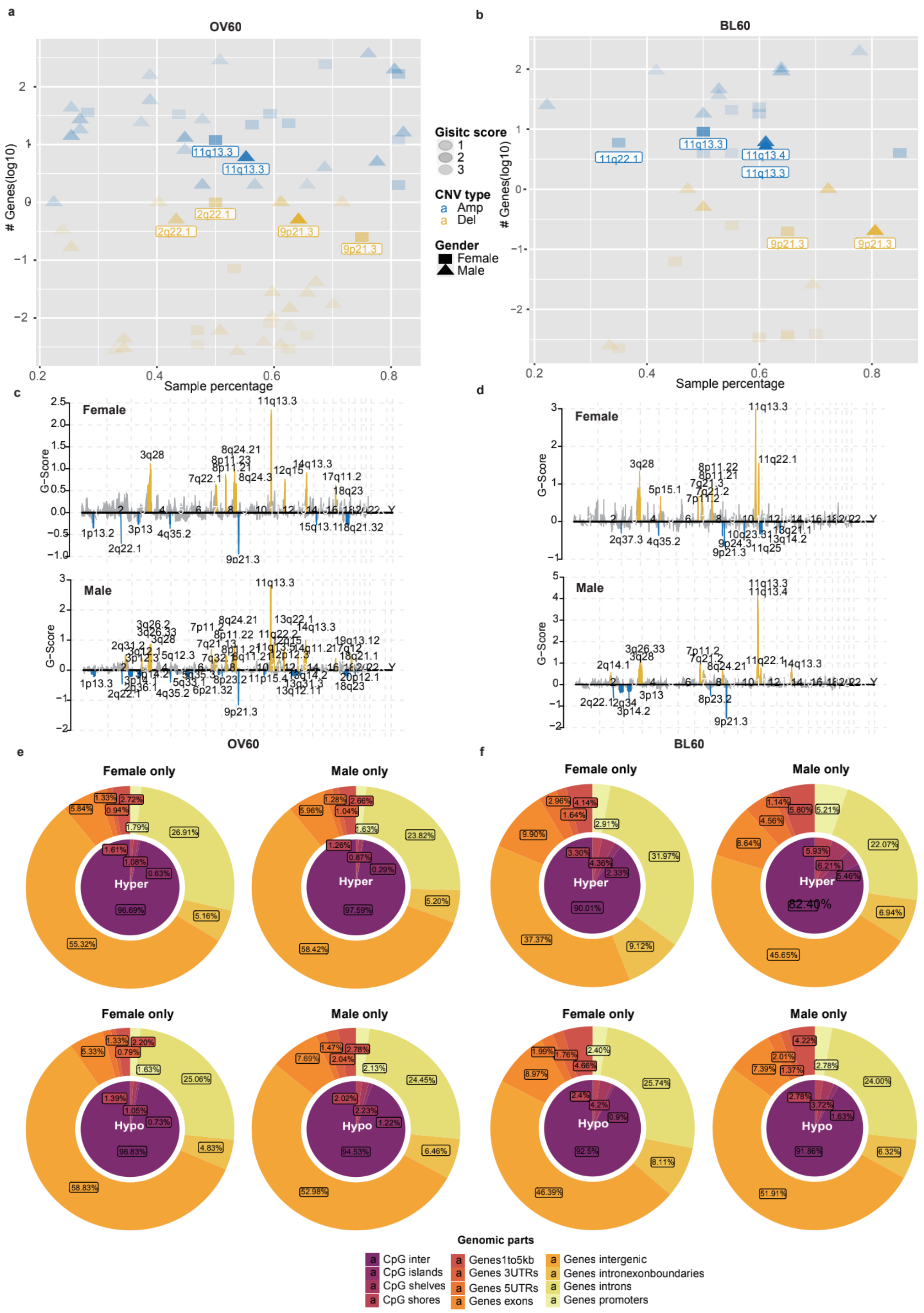

Figure S3 CNV and methylation in different sex groups and age groups. (a) Patients over 60 years old and (b) patients below 60 years old plot of significantly altered cytobands regarding the number of samples in which it is altered and the number of genes it contains. The transparency of each bubble is according to the -log10 transformed G-score. The top 3 cytobands with the highest G-score in each sex are

labeled. Patients over 60 years old (c) and patients over 60 years old (d) female and male genomic plots with segments highlighting significant amplification and deletion regions. (e) Distributions of gene parts and CpG regions in patients over 60 years old and (f) patients below 60 years old. Both age groups had a female-only subgroup and a male-only subgroup. Hypermethylation and hypomethylation were also shown for each subgroup.

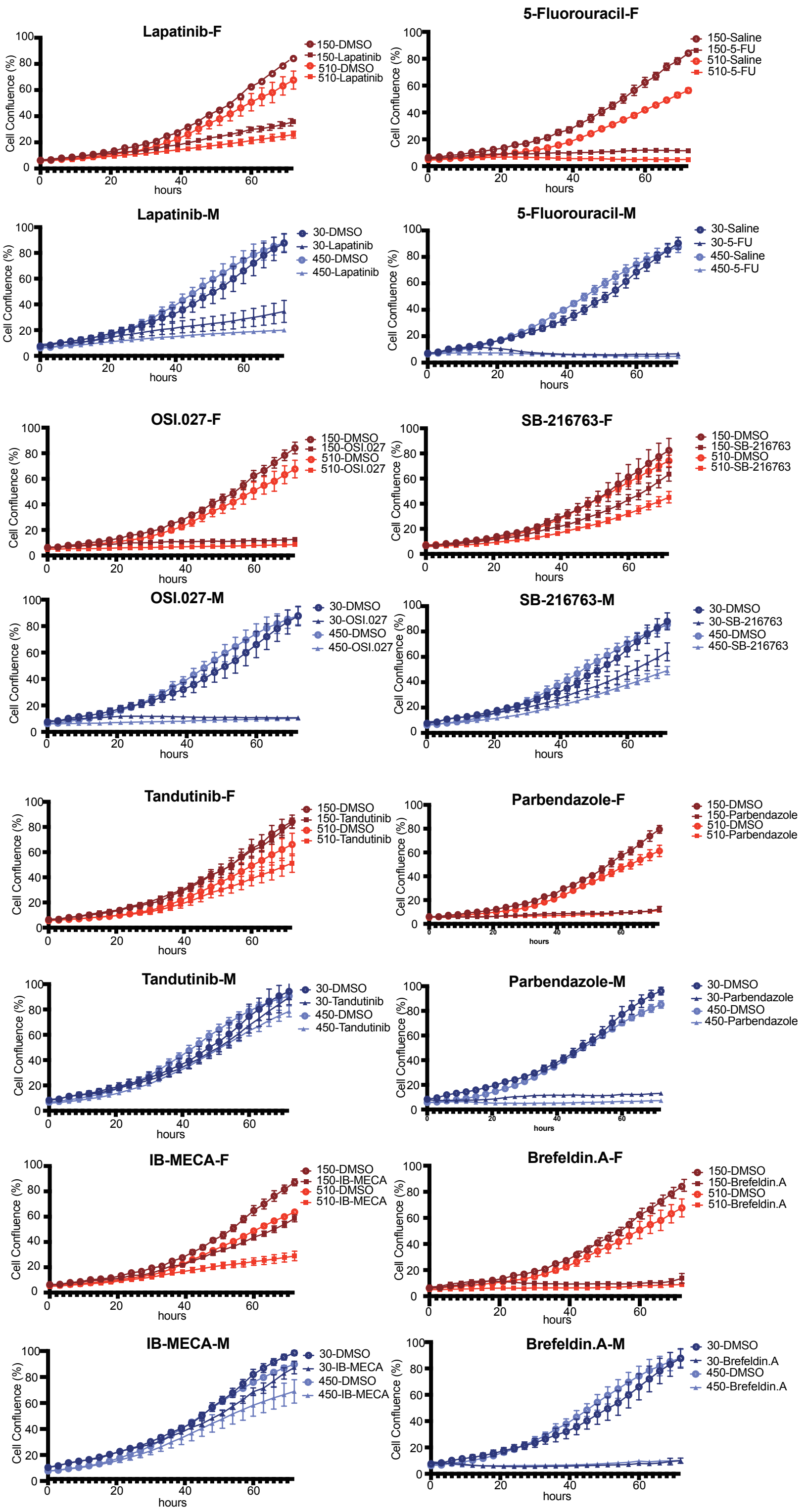

Figure S4 Proliferation rate of 8 other candidate drugs that potentially have sex-biased sensitivity to ESCC patients. Growth curves of KYSE150, KYSE510, KYSE30 and KYSE450 cells measured by IncuCyte S3 for 72 h. Cells were treated with the indicated drugs at different concentrations. Lapatinib, 1 μM; 5-fluorouracil,

2 μM; OSI.027, 1 μM; SB-216763, 10 μM; Tandutinib, 1 μM; Parbendazole, 10 μM; IB-MECA, 40 μM; Brefeldin.A, 200 nM.
